# Effect of simulated gastro-duodenal digestion on the allergenic reactivity of beta-lactoglobulin

**DOI:** 10.1186/2045-7022-1-6

**Published:** 2011-08-09

**Authors:** Apostolos Bossios, Maria Theodoropoulou, Lucie Mondoulet, Neil M Rigby, Nikolaos G Papadopoulos, Hervé Bernard, Karine Adel-Patient, Jean-Michel Wal, Clare EN Mills, Photini Papageorgiou

**Affiliations:** 1 Athens University, P. & A. Kyriakou Children's Hospital, Athens, Greece; 2 Lab d'Immuno-Allergie Alimentaire, INRA-CEA, Saclay, France; 3 Institute of Food Research, Norwich Research Park, Colney NR4 7UA UK; 4 Krefting Research Centre, Department of Internal Medicine, Institute of Medicine, The Sahlgrenska Academy, University of Gothenburg, Gothenburg, Sweden; 5 DBV Technologies, 92220, Bagneux, France; 6 Thessaly University Hospital, Larissa, Greece

**Keywords:** in vitro digestion, cow's milk allergy, β-lactoglobulin, flow cytometry, Basophil activation, skin prick test

## Abstract

**Background:**

Cow's milk (CM) allergy affects about 2% of infants. The allergenicity of dietary proteins, including those from CM, has been related to their digestibility although the generality of the link and its causality remains to be demonstrated. In this study we use an in vitro digestion system, to investigate the digestibility of β-lactoglobulin (blg) during gastrointestinal transit and to assess the impact of this process on blg allergenic reactivity in CM allergic children.

**Methods:**

Blg digesta were prepared using an *in vitro *digestion protocol simulating either gastric digestion alone or followed by duodenal digestion with or without phosphatidylcholine (PC). Biochemical analysis of blg digesta was performed by SDS-PAGE and their concentration was measured by a sandwich ELISA. Assessment of their allergenic reactivity was done *in vitro *by EAST inhibition, specific basophil activation (basotest) and lymphocyte proliferation (PCNA-flow cytometry) assays using sera and cells from patients allergic to blg and *in vivo *by skin prick testing (SPT) of these patients.

**Results:**

Blg was only broken down to smaller peptides after gastro-duodenal digestion although a sizeable amount of intact protein still remained. Digestion did not modify the IgE binding capacity of blg except for gastro-duodenal digestion performed in the absence of PC. These results are consistent with the quantity of intact blg remaining in the digesta. Overall both gastric and gastroduodenal digestion enhanced activation of sensitized basophils and proliferation of sensitized lymphocytes by blg. However, there was a tendency towards reduction in mean diameter of SPT following digestion, the PC alone during phase 1 digestion causing a significant increase in mean diameter.

**Conclusions:**

Digestion did not reduce the allergenic reactivity of blg to a clinically insignificant extent, PC inhibiting digestion and thereby protecting blg allergenic reactivity. SPT reactivity was reduced compared to blg immunoreactivity in *in vitro *tests.

## Background

Cow's milk allergy (CMA) is defined as an immunologically mediated adverse reaction to cow's milk proteins [1,2]. In industrialized nations, CMA affects approximately 2% of infants under 2 years of age, and is one of the most common food allergies in this age group [3-6]. In the majority of cases, allergic reactions to cow's milk proteins amongst children are thought to be IgE-mediated [7,8]. It also occurs in adults although the prevalence is unknown. CMA presents with a broad range of clinical symptoms and syndromes, ranging from acute anaphylactic manifestations to diverse disorders, such as urticaria, angioedema, atopic dermatitis, food-associated wheeze, infantile colic, gastro-oesophageal reflux (GOR), oesophagitis, cow's milk enterocolitis, food-associated proctocolitis and constipation [1,4].

The globular protein β-lactoglobulin (blg) is present in the whey fraction of the milk of most mammals, but not in human milk [9]. A member of the lipocalin superfamily, native blg exists as a *M_r _*36,000 dimer at neutral pH comprising identical subunits, which adopt a β-barrel structure with a lipid-binding calyx stabilized by two intra-molecular disulphide bonds. Such structural features are thought to contribute to the stability of this protein to, for example, proteolysis [10]. The relative resistance of blg to acid hydrolysis as well as to proteases may allow some of the protein to escape gastrointestinal digestion. This increases the probability that intact blg will be absorbed through the gut mucosa and may explain why it is one of the most potent allergens in cow's milk [11,12]. There is limited information regarding the effect of digestion on immunological characteristics of blg, including its effect on IgE binding and T-cell stimulatory antigenic epitopes. Recent information regarding impaired digestion seems to facilitate sensitization of individuals not allergic to milk. Fragmentation of blg using a combination of chemical and enzymatic means has been used to identify the major IgE epitopes of this protein and indicates that fragments of the protein retain their IgE binding capacity [13]. Cellular and immunological techniques have also been employed in order to evaluate the residual immunogenicity of hydrolyzed milk formulas and showed that residual peptides have a reduced IgE binding capacity and either caused diminished skin reactions [14,15] or had reduced immunogenic properties at the T-cell level [16].

However, the degree to which sensitization occurs towards intact versus hydrolysed allergens remains unknown and yet such knowledge is essential if we are to gain a deeper understanding of the mechanisms of food allergy.

Stability to gastric digestion has been proposed as one of the properties shared by food allergens [11,17,18]. The use of digestion stability as a criterion for protein allergenic reactivity assessment stems from the general belief that for a protein to elicit an allergic response, it must survive the acid and proteolytic environment of the human gastrointestinal system to reach and be absorbed through the intestinal mucosa [11,19] and be recognised by the immune system. Numerous food allergens have been shown to be stable to conditions simulating human gastrointestinal digestion [20-23]. However, several recent investigations do not support the view that food allergens are necessarily more resistant to digestion than are non-allergenic proteins [24-26]. This has fuelled the debate as to whether resistance to digestion [18,27] should be used as one of the main criteria in assessing the potential risks of allergenic reactivity posed to consumers by novel food and genetically modified organisms (GMOs) especially since we have an incomplete understanding of its role in the pathogenesis of food allergy [20,28-30].

The aim of the present study was to investigate the effect of gastrointestinal digestion on blg allergenic reactivity and its impact on the ability of blg to elicit an allergic response in CMA children. An *in vitro *digestion model was employed which mimics the conditions of the human gastro-intestinal system as it incorporates physiological surfactants such as gastric phosphatidyl choline and a model mixture of duodenal bile salts [21,31]. The residual IgE binding capacity of blg digesta was assessed using human allergic sera from CMA allergic children. Since allergens and their digestion products may affect the cells of the immune system by a variety of mechanisms, we employed three different methods, two *in vitro *(assessing the ability of digesta to activate basophils and stimulate lymphocyte proliferation) and one *in vivo *(skin testing), in order to evaluate the cellular responses of CMA children to blg digesta.

## Materials and methods

### Beta-lactoglobulin (blg)

Bovine blg from milk was purchased from Sigma Chemical Co. (St. Louis, Mo, USA) in the purest form available as lyophilized powder and dissolved in double distilled water to a concentration of 5 mg/ml. It comprised a mixture of variants A and B.

### Production of *in vitro *blg digesta

An *in vitro *digestion system was employed, which comprises a phase 1 digestion analogous to the gastric phase of human digestion and a phase 2 which mimics transfer into the duodenal compartment. Digestion of blg was carried out essentially as described by Moreno et al 2005. Briefly phase 1 (gastric) digestion was performed using 5 mg/ml blg dissolved in 0.15 M NaCl, pH 2.5 in the presence or absence of hen egg phosphatidyl choline (PC) vesicles (final concentration of 6.3 mM) and pepsin (Sigma-Aldrich, St. Louis, Mo, USA pepsin A; EC 3.4.23.1; ~3, 5 ku/mg protein) to give a final enzyme: substrate ratio (E:S) of 1:20 (w:w). Gastric digestion was then performed at 37°C for 2 h with shaking and terminated by the raising the pH to 7.5 by the addition of 40 mM ammonium bicarbonate. Prior to phase 2 digestion the pH of gastric digesta was re-adjusted to 6.5 by the addition of 1 M NaOH and the following added to a final concentration of 0.1%, (w:v) porcine pancreatic lipase, porcine colipase (0.055%, w:v), 5.8 mM PC, 2.3 mg/ml blg, 7.4 mM bile salts, 9.2 mM and 24.7 mM Bis-Tris. Approximately physiological ratios of blg (as denoted by the initial concentration in phase 1) and proteases (protein: trypsin: bovine chymotrypsin) of 400:4:1 (w:w:w) were added. Enzymes were all purchased from Sigma-Aldrich,(Dorset, UK). Digestions were incubated at 37°C for 15 min to 2 h with shaking and terminated by the addition of soybean Bowman-Birk trypsin-chymotrypsin inhibitor (Sigma Dorset, UK) at a concentration calculated to inhibit twice the amount of trypsin and chymotrypsin present in the digestion mix. All digesta were provided as frozen solutions for allergenic reactivity assessment, aliquoted and stored at -70°C prior to use.

### Characterization of digesta

#### SDS-PAGE

Biochemical characterization of digested blg samples was performed by means of SDS-PAGE according to the method of Laemmeli [32] using Coomassie brilliant blue staining (CBB). The molecular weight was calculated by using prestained protein standards (Broad Range Protein Molecular Weight Markers, Promega). Samples from digesta were loaded on 18% polyacrylamide Tris-glycine gels and were electrically separated. The gels were fixed for 5 min in 5% TCA, washed for 2 h with SDS Wash (45.5% (v/v) methanol, 9% (v/v) acetic acid), stained for 10 min with CBB staining solution (0.1% (w/v) Coomasssie Brilliant Blue R250, 15% (v/v) methanol, 10% (v/v) acetic acid) and destained with 25% (v/v) methanol and 7.5% (v/v) acetic acid. The stained gel images were analyzed by using Image Gauge V3.1 (Fuji Film, Tokyo, Japan).

### Quantitation of blg digesta

#### ELISA

Blg was quantified using a sandwich ELISA assay as described by Negroni et al. [33]. Briefly, assays were performed in 96-well microtiter plates coated by passive adsorption with 100 μl of the first antibody MAb BLG-97N (capture antibody at a concentration of 5 μg/ml). After extensive washings and saturation, plates were sealed and stored at +4°C. Fifty microliters of blg standard or samples, and 50 μl of tracer, which consist of a second MAb BLG-117N labelled with acetylcholinesterase (AChE) were then added. After 18 h of reaction at 4°C, the plates were washed and 200 μl Ellman's reagent was used as enzyme substrate and absorbance was measured at 414 nm. Detection limits of 30 pg/ml were obtained for blg with negligible cross-reactivity towards other milk proteins or fragments of blg.

### Cow's Milk Allergic Subjects and *in vivo *assessment of the allergenic reactivity of blg digesta

Specific sera and blood samples were obtained from P&A Kyriakou Children Hospital, 2^nd ^department of Pediatrics. In total 20 patients aged 7 months to 7 years (mean age 2.4 yrs), 11 female and 9 male were included in this study. IgE-mediated allergy to cow's milk was diagnosed on the basis of detection of specific IgE antibodies (≥ 3 class or > 4.0 KUA/L) in the CAP- FEIA System (Phadia, Uppsala, Sweden), positive skin test reactivity and positive oral provocation test results. These patients provided the source of IgE specific blg sera, sensitized basophils and peripheral blood lymphocytes (PBL). In the same patients skin prick tests (SPT) were performed with intact and purified blg digesta. Ethics Committee approval, as well as written informed consent from each subject before entry into the study, was obtained. Following the completion of *in vitro *testing of gastric (phase 1) and gastro-duodenal (phase 2) digesta, concentrations that were not cytotoxic were used in skin testing. SPTs were performed on the flexor aspect of the forearm with a standardized prick needle. Histamine dihydrochloride (10 mg/ml) was used as a positive control and the prick solution (Soluprick^®^, ALK, Hørsholm) which contains glycerol and 0, 9% (w/v) NaCl as a negative control. Patients were tested with freshly prepared sterile (0.2 μm) filtered solutions of native blg (internal control, dissolved to 500 μg/ml in phosphate buffered saline [PBS]) and blg digesta diluted 1:10 (v/v) with PBS. Reactions were recorded after 15 min. A wheal with a mean diameter of > 3 mm was considered positive [34].

### *In vitro a*ssessment of allergenic reactivity of the blg digesta

#### EAST inhibition assay

Analysis of the immunoreactivity of the blg digesta was performed by EAST inhibition with human sera from milk allergic patients. Microtiter plates were coated with an in-house anti-human IgE monoclonal antibody (i.e. LE27) and 100 μl per well of diluted patient serum was distributed across each plate and incubated overnight at +4°C. After washing, 50 μl per well each of inhibitor and tracer were dispensed and plates were then incubated at room temperature for 4 h. Increasing concentrations of blg from gastric and gastro-duodenal digesta in the presence or absence of PC served as inhibitors. Enzymatic tracer was prepared by covalent linkage of blg to the tetrameric form of acetyl cholinesterase (AChE) as previously described for other protein tracers [35]. After further plate washing, Ellman's reagent was added as the enzyme substrate as described for the blg ELISA above. IgE binding was expressed as B/B_0 _where B_0 _and B represent A414 values obtained for blg tracers bound to immobilized IgE in the absence (B_0_) or presence (B) of a known concentration of inhibitor.

#### Basophil Activation test (Basotest)

The Basotest (Becton-Dickinson BD, San Jose, Calif., USA) was employed to assess the response of CM sensitized basophils to digesta [36,37]. This is a binding technique based on the activation and the expression of CD63 (gp53) by the sensitized basophils in the presence of allergens. The test was performed according to the manufacturer's instructions. In brief, samples were incubated for 20 min at 4°C with 20 μl of PE-anti IgE antibody and anti-FITC-gp53. Erythrocytes were subsequently removed by the addition of 2 ml of lysing solution (BD). Cells were washed twice with PBS solution, resuspended in 200 μl of PBS and then analyzed within 1 h with a FACSsort flow cytometer (Becton-Dickinson BD, San Jose, Calif., USA). The basophil population was gated by the expression of PE anti-IgE. The expression of CD63 (gp53) was analyzed on this gated population. The data acquisition was generally carried out on 1000 basophils. The results of basophil activation were expressed as an Activation Index (AI) representing the ratio between the percentage (%) of cells expressing CD63 on their surface incubated in the presence of digesta and the percentage (%) of cells expressing CD63 incubated in the absence of digesta.

A percentage of > 15% is considered positive according to the manufacturer's instructions.

### In vitro proliferation of sensitized PBLC

#### Cell cultures

PBMC were obtained from whole blood by Ficoll-Hypaque (Pharmacia, Uppsala, Sweden) density centrifugation. Cells were washed twice with Hank's Balanced Salt Solution (HBSS, Gibco, Grand Island, NY, USA) and resuspended in complete culture medium comprising of RPMI-1640 with Glutamax, 10% (v/v) heat inactivated human AB serum, 50 μg/ml gentamycin and 10 mM Hepes buffer (Gibco Grand Island, NY, USA) at a final concentration of 10^6 ^cells/ml. Cells were put into culture in a humidified 5% CO_2 _incubator at 37°C for 6 days with or without the addition of 10 μg/ml of native blg (Sigma-Aldrich, St. Louis, Mo, USA) and with the digesta described above at a concentration of 10 μg/ml, determined by preliminary dose-response experiments.

#### Lymphocyte proliferating cell nuclear antigen expression analysis (PCNA)

PCNA analysis was employed to assess the response of CM sensitized lymphocytes to digesta [38]. Cells were initially permeabilized in a buffer comprising of 0.2 mg/ml Na_2_HPO_4_·2H_2_O, 1 mg/ml KH_2_PO_4_, 45% (v/v) acetone and 9.25% (v/v) formaldehyde (Sigma-Aldrich, St. Louis, Mo, USA). Permeabilized cells were immediately stained with 10 μl of an anti-PCNA, fluorescein-conjugated monoclonal antibody (Pharmingen, San Diego, Calif, USA) for 15 min at RT. They were then washed twice, fixed with 1% paraformaldehyde in PBS and counted with a FACSsort flow cytometer (Becton-Dickinson BD, San Jose, Calif., USA). Fluorescence data were collected on 10^4 ^cells and histogram analysis of green fluorescence (FL-1) was performed with the use of Cell Quest software. The results of lymphocyte proliferation were expressed as a Proliferation Index (PI) representing the ratio between the percentage (%) of cells expressing PCNA on their surface cultured in the presence of digesta and the percentage (%) of cells expressing PCNA cultured in the absence of digesta

A proliferation index of > 2 is considered significant.

### Statistics

All data were analyzed using SPSS software 11.5. For statistical analysis we used Student's t-test for paired data, 2-tailed p values. A p value of < 0.05 was considered statistically significant.

## Results

### Characterization of blg digesta

**Figure 1:**
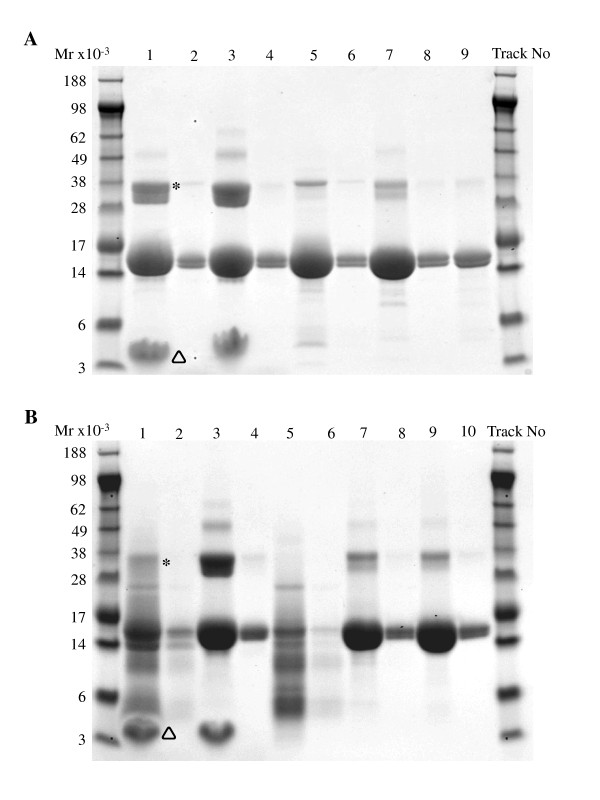
**Characterization of blg digesta**. A) phase 1 digestion: Lane 1 BLG @ 5 mg/ml, +PC (phosphatidyl choline) +E (enzyme). Lane 2 BLG @ 0.5 mg/ml, +PC +E. Lane 3 BLG @ 5 mg/ml, +PC -E. Lane 4 BLG @ 0.5 mg/ml, +PC -E. Lane 5 BLG @ 5 mg/ml, -PC +E. Lane 6 BLG @ 0.5 mg/ml, -PC +E. Lane 7 BLG @ 5 mg/ml, -PC -E. Lane 8 BLG @ 0.5 mg/ml, -PC -E. Lane 9 BLG @ 5 mg/ml. B) phase 1+2 digestion: Lane 1, BLG @ 5 mg/ml, +PC (phosphatidyl choline) +E (enzyme). Lane 2 BLG @ 0.5 mg/ml, +PC +E. Lane 3 BLG @ 5 mg/ml, +PC -E. Lane 4 BLG @ 0.5 mg/ml, +PC -E. Lane 5 BLG @ 5 mg/ml, -PC +E. Lane 6 BLG @ 0.5 mg/ml, -PC +E. Lane 7 BLG @ 5 mg/ml, -PC -E. Lane 8 BLG @ 0.5 mg/ml, -PC -E. Lane 9 BLG @ 5 mg/ml. Lane 10 BLG @ 0.5 mg/ml. Asterisk (*) denotes BLG SDS resistant dimmer, triangle (Δ) denotes Phosphatidyl choline.

SDS-PAGE analysis of gastric digestion products showed the presence of a single protein band of *M_r _*18,500 corresponding to that of intact native blg indicating the protein was essentially undigested blg even after 2 h gastric digestion. During subsequent duodenal digestion for 15 min the protein was broken down into a series of lower *M_r _*polypeptides although a sizable amount of intact protein still remained (Figure 1). The 15 min time point was chosen as approximately the time it might take gastric digesta to transfer to the duodenal compartment and flow down the small intestine to around the position of the first Peyer's patch, a relevant site where immunologically relevant sampling of the luminal contents of the gut might occur.

In order to quantify the amount of intact blg remaining in digesta they were analysed with an ELISA specific for intact native protein. Table 1 shows that there was a slight reduction in intact blg in all gastric digesta, with around 40% of the protein apparently being broken down. However, following gastro-duodenal digestion only around 3% of intact blg remained, although the inclusion of PC in the gastric digestion appeared to completely protect the protein from degradation in the duodenal environment for at least 15 min.

**Table 1:** Levels of intact native blg concentrations in different digesta determined by sandwich ELISA

		Intact blg (mg/ml)		Residual blg (as % of control)	
	
Digestion		Control	Enzyme	Control	Enzyme
**Gastric**	**-PC**	5.0	3.4	100	70
**(phase 1)**	**+PC**	4.7	3.2	100	68

**Gastroduodenal**	**-PC**	5.0	0.14	100	3
**(phase 1+2)**	**+PC**	4.5	3.0	100	66

Control digestions were performed without the addition of proteases.

### *In vitro *assessment of allergenic reactivity of digesta

**Figure 2:**
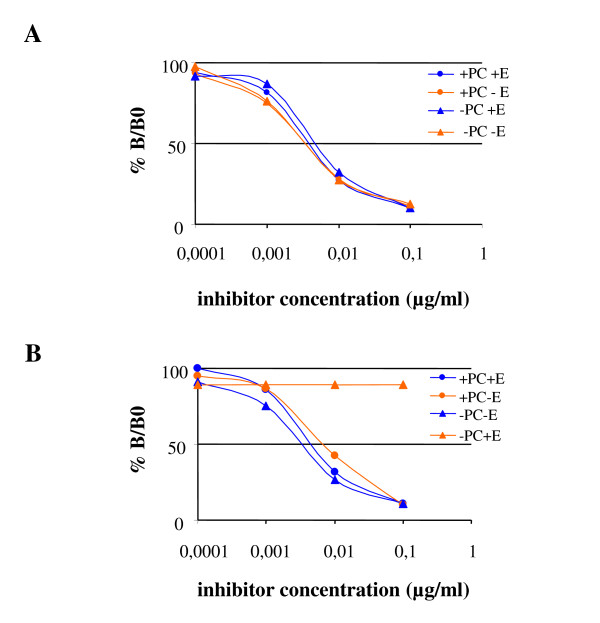
**IgE immunoreactivity of blg digesta**. Analysis of the immunoreactivity of blg digesta by EAST inhibition using serum from a CM allergic child. A: phase 1, B: phase 1+2.

Figure 2 shows a typical EAST inhibition curve obtained with sera from a single CMA child. Gastric digestion had no effect on IgE binding, the same 50% binding inhibition doses (IC_50_) of 5 ng/ml being obtained for digesta and control incubations performed in the absence of proteases, for all sera analysed. For gastro-duodenal digestion in the absence of PC no inhibition of binding was observed at the concentrations employed (Figure 2) indicating that the IC_50 _value was greater than 10 μg/ml. In contrast gastro-duodenal digestion in the presence of PC did not alter the IC_50 _values which were of the order of 5 ng/ml for all the digesta, including the controls 10 μg/ml.

Having established the IgE reactivity of digesta, their ability to activate basophils was assessed. The percentage of activated basophils was determined with the Basotest to further investigate the properties of digested and undigested extracts as elicitors of allergic reactions. Basotest results are given as the percentage of basophils expressing CD63. The specificity of the test was confirmed with a non-sensitizing allergen and none of the CMA patients showed basophilic activation of > 15% (2.0-6.5%). Non-allergic subjects exhibited no basophilic activation with values 2.1-3.5%. A high background was observed in a few instances with cells from highly allergic children which were excluded from the study, although upon exposure to the specific allergen, basophils from these children showed a marked degree of additional basophilic activation which was significant once the high background was subtracted (results not shown). Despite this observation for the purpose of this study, only basophils from patients with low activation background were employed as target cells.

**Figure 3:**
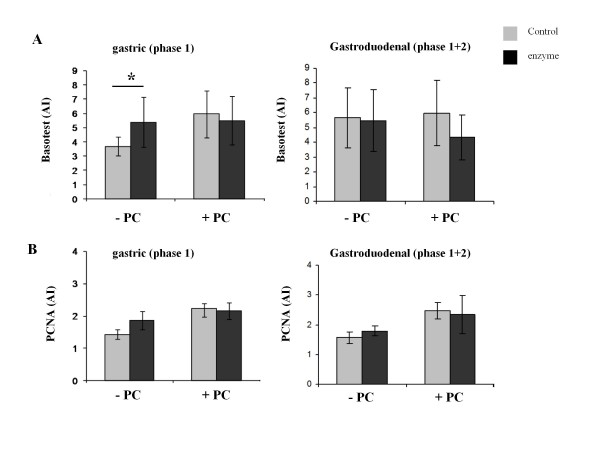
***In vitro *assessment of allergenic reactivity of blg digesta**. Effect of gastric (phase 1) and gastro-duodenal (phase 1+2) digestion on the ability of blg to activate basophils (A) or stimulate proliferation of PBLCs (B) from CMA children. Results are expressed as mean values ± SEM. AI: Activation Index representing the ratio between the percentages (%) of cells expressing CD63 on their surface incubated in the presence of digesta and the percentage (%) of cells expressing CD63 incubated in the absence of digesta. PI: Proliferation Index representing the ratio between the percentage (%) of cells expressing PCNA on their surface cultured in the presence of digesta and the percentage (%) of cells expressing PCNA cultured in the absence of digesta. PC:phosphatidylcholine. * P < 0.05.

All the blg digesta were able to activate IgE sensitized basophils in all thirteen (13) CM allergic children examined with a statistically significant difference between samples observed only when blg was subjected to gastric digestion in the presence of PC (p = 0,015) (Figure 3A). The presence of PC alone in the gastric digestion mix did appear to activate basophils to some extent, although the difference was not statistically significant compared with the digestion buffer alone.

Lastly the effect of digestion on the ability of blg to stimulate lymphocyte proliferation in CMA individuals was assessed using PCNA expression. In healthy individuals PCNA expression is < 5% of cells cultured in the presence of blg while patients with CMA and sensitive to the specific allergen (in this case blg) gave PCNA levels of > 10%.

All blg digestion samples were able to stimulate proliferation of lymphocytes from CMA patients. The presence of PC vesicles in the control gastric digestion mix (to which no enzymes had been added), increased the lymphocyte response to blg, although addition of the gastro-duodenal mix (containing bile salts able to disrupt the PC vesicles) abolished this effect. The gastro-duodenal mix without PC increased the ability of blg to activate lymphocytes, possibly due to the action of the complex mix of surfactants it contains on the lymphocytes. Despite these effects a comparison on the effect of gastric or gastro-duodenal digestion of blg with these respective control samples showed that digestion did not diminish the lymphocyte proliferative responses towards blg (Figure 3B).

### *In vivo *assessment of purified allergen digesta allergenic reactivity

#### Effect on skin prick reactivity

**Figure 4:**
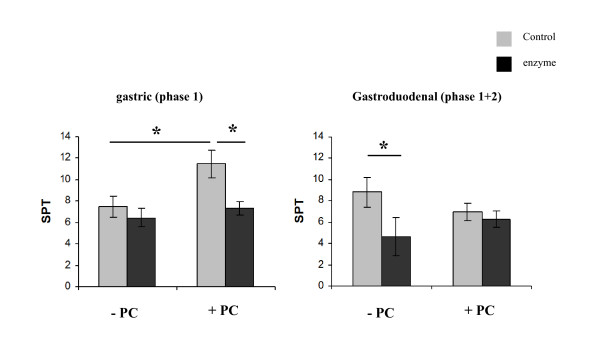
***In vivo *assessment of allergenic reactivity of blg digesta**. Effect of phase 1 and phase 1+2 blg digestion on SPT reactivity of CM allergic children. Results are expressed as mean values ± SEM. PC:phosphatidylcholine. * P < 0.05.

Skin prick testing with filtered digestion samples and controls was performed in 5 patients with CMA. The results suggested a tendency towards reduction in the mean diameter of skin prick test following both gastric and gastro-duodenal digestion although even the latter did not reduce it to clinically insignificant levels. However, digestion in the presence of gastric PC resulted in a significant increase in the mean wheal diameter for gastric digestion, although it was diminished following gastro-duodenal digestion (Figure 4). The PC itself was not found to cause any skin reaction in controls (data not shown).

**Figure 5:**
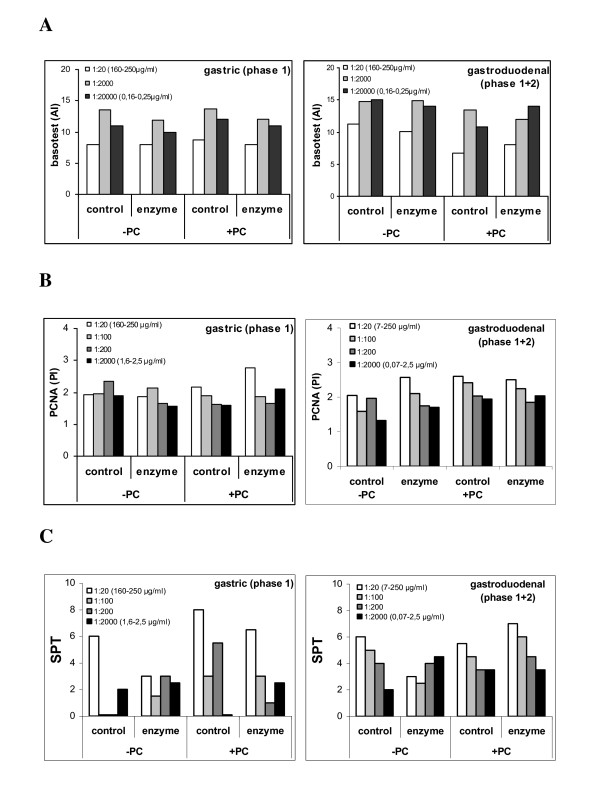
**Titration of in vitro produced blg digesta**. An assessment of dose-responses of in vitro produced phase 1 and phase 1+2 blg digesta and their effect on basophil activation (A), PCNA reactivity (B) and SPT reactivity (C) of CM allergic children. AI and PI as indicated in figure 3.

In order to evaluate the allergenic potency of the blg digesta and controls *in vivo*, an assessment of dose-responses of digesta on basophil activation, lymphocyte proliferation and SPT reactivity of patients allergic to cow's milk was made by titration using a serial of the various samples (Figure 5). The results are illustrated in Figure 5 and indicate that the digestion had little impact on three different measures of the allergenic activity of blg.

## Discussion

The aim of the present study was to determine the effect of gastric and duodenal digestion on the biochemical, immunochemical and allergenic characteristics of blg protein, one of the principal allergens responsible for IgE-mediated CMA. Many allergens, but not all, are stable to the extremes of pH and proteases encountered in the mammalian stomach and small intestine [19,22,39-41] and this has led to much debate about the importance of stability to determining the allergenic potential of food proteins. Many of the studies carried out on stability of allergens to digestion have used conditions, such as substrate: protease ratios which are far from those found *in vivo *in the gastro-duodenal environment or do not take into account the effect of pre-processing by mastication and swallowing. Another factor which has been neglected is the effect that physiological surfactants, such as gastric PC and duodenal bile salts, may have on stability of proteins to digestion. We used an *in vitro *system which takes such factors into account and attempts to model the *in vivo *human process more closely.

As described before, blg was found to be almost completely resistant to breakdown in the gastric compartment, reflecting the resistance of this protein to pepsinolysis [24,42]. However, the presence of the physiological surfactant PC in the gastric phase of digestion was found to protect the blg from breakdown in the duodenal environment, in marked contrast to the susceptibility of the protein to trypsin and chymotrypsin in absence of PC. Other studies performed with simulated gastric or intestinal fluid have also shown that blg was almost completely digested by pancreatic enzymes [24,42]. These data indicate that blg is resistant to both gastric and duodenal digestion when physiologically relevant levels of PC are included in the digestion mix. The mechanism underlying the protective effect of PC on blg breakdown in the duodenal environment is not clear but may relate to the ability of blg to bind lipids, ligand binding stabilising proteins [10] or interactions of a subtle nature with lipids as have been found to play a role in the protective effect of PC on the digestion of another cow's milk allergen, α-lactalbumin [31]. These results indicate the limitations of using the results of the published protocols for resistance to pepsin digestion as indications of potential allergenic activity and emphasise the importance of including physiologically relevant surfactants in the digestion mix for such studies.

Assessing the digestibility of a protein is not by itself a sufficient measure of the allergenic potential of a protein and hence we also investigated the effects of digestion on various measures of the allergenic activity of blg and compared the properties of digested and undigested extracts as elicitors of allergic reactions. Indeed, there are some intriguing indications that the inherent stability of proteins and the characteristics of peptide antigens displayed to antigen presenting cells have an impact in various and sometimes contradictory ways on the type of induced immune responses which has clinical indications [43-46]. Due to the diversity of immune mechanisms leading to food allergy, a variety of procedures for predicting allergenic reactivity of food antigens has been developed.

Using human CMA sera the residual IgE binding capacity of blg after digestion was found to be entirely consistent with the levels of residual native protein found in the digesta. Thus, IgE binding was only reduced following gastro-duodenal digestion in the absence of PC, indicating that under these conditions digestion either modified the IgE epitopes on blg in such a way that they were recognised a much lower affinity than the intact protein, or that digestion has destroyed many of the epitopes.

In contrast, even though gastro-duodenal digestion of blg in the absence of PC resulted in a dramatic loss of intact native protein and IgE reactivity as judged by EAST inhibition there was still sufficient intact blg and/or fragments to activate IgE sensitized basophils from blg allergic patients. Similarly both gastric and gastro-duodenal digestion, in the presence or absence of gastric PC, did not abolish the ability of blg to induce proliferation of lymphocytes from CMA individuals. These findings suggest that following gastric and duodenal digestion blg maintains its IgE and T-cell reactivity. In this study the presence of PC was found to enhance T-cell reactivity in both digestion phases and IgE reactivity only after phase 1 digestion.

The ability of blg digesta to activate basophils and stimulate lymphocyte proliferation (Table 1, Figure 3) was not significantly correlated with each other or the concentration of residual blg in the digesta. However, this analysis did demonstrate that overall digestion enhanced activation of sensitised basophils and proliferation of sensitized lymphocytes without reaching statistical significance. Blg showed reduced skin reactivity following gastric digestion, although the PC vesicles in the digestion mix increased the potency of undigested blg. PC alone did not cause skin reaction in control non CM allergic individuals. The effect of PC was abolished by the addition of bile salts in the gastro-duodenal mix. These observations may result from the PC vesicles presenting the blg in a more potent form in skin testing after phase 1 digestion, which decreased after phase 1+2. Why there is a divergence between the SPT results and the Basotest is not clear. Perhaps SPT is more complex and sensitive.

In comparing the effect of blg digesta on SPT reactivity in relation to the blg concentration, a correlation was found between SPT reactivity and residual intact blg concentration in all digesta (Figure 4) apart from the gastro-duodenal digesta prepared in the absence of PC where intact blg concentrations were very low but much of the SPT reactivity was retained. Also, in phase 1 it was significantly reduced. Interestingly, no such effect of the presence of PC in phase 1+2 was noted. These findings indicate that the presence of PC inhibits blg digestion resulting in increased allergenic reactivity of blg as assessed by skin prick reactivity only. Titration of the various digesta by serial dilutions showed that they were highly allergenic, even at very low concentrations in the SPT analysis. These data are not entirely consistent; this is not in agreement with the *in vitro *cellular assays, where only a small effect of the PC effect was less clearly found. Perhaps the skin prick test SPT is a more sensitive means of assessing allergenic reactivity, while the *in vitro *techniques may require further refinement and validation. Furthermore, despite the dramatically reduced blg concentration in phase 1+2 the allergenic reactivity as assessed by basotest, PCNA and SPT was not affected. Of course, considering the small size of our population we cannot exclude the possibility that larger population might have given us the possibility to detect any small differences that perhaps are now overlooked. However, our purpose here was to detect any big differences that could affect the clinical praxis, and which could be shown through our pilot study population. No gross differences were seen, arguing that our results can be translated in the clinical routine.

As assessed by the methods employed in this study *in vitro *digestion has a heterogeneous effect on the ability of blg to elicit reactions in vivo and in vitro, one of the major milk proteins: it may leave it unaltered, reduce or enhance it. It also suggests that the residual blg surviving simulated gastrointestinal digestion still contains sufficient immunologically active structures (T-cell and B-cell epitopes) to potentially either sensitise an individual or elicit an allergic reaction, and possibly sensitise. Allergenic activity might be attributed to inactivation or destruction of epitope structures, formation of new epitopes, or improved access of previously hidden epitopes. This also highlights that the nature, structure and biological activity of peptides may be completely different when these are produced from a purified protein in a buffer solution (e.g. in a pepsin resistance test) to those produced in a complex biological medium, where interactions with other constituents may occur.

## Conclusions

Our data indicate that in assessing the impact of digestion on food protein allergenic reactivity more than one parameter should be taken in to consideration and digestibility should be assessed using several endpoints. Thus the estimation of digestive stability is crucial, but the optimal form of the assay for assessing allergenic activity is also important.

## Competing interests

The authors declare that they have no competing interests.

## Authors' contributions

Conceived and designed the experiments: PP, ENCM, JMW. Analyzed the data: AB, MT, LM, KAP, NMR Contributed reagents/materials/analysis tools: NGP, HB, JMW, ENCM. Wrote the paper: PP, MT, AB. All authors read and approved the final manuscript.
